# Rare type I *CBFβ*/*MYH11* fusion transcript in primary acute myeloid leukemia with inv(16)(p13.1q22): a case report

**DOI:** 10.1590/1414-431X2021e11605

**Published:** 2021-10-29

**Authors:** Wenyi Zhang, Hainan Wang, Peilei Zhang, Hongliang Li, Xiaoli Ma, Hongxing Liu

**Affiliations:** 1 Department of Clinical Laboratory, Traditional Chinese Medical Hospital of Langfang City, Langfang, Hebei, China; 2 Department of Medical Hematology, Traditional Chinese Medical Hospital of Langfang City, Langfang, Hebei, China; 3 Division of Pathology and Laboratory Medicine, Hebei Yanda Lu Daopei Hospital, Langfang, Hebei, China

**Keywords:** Acute myeloid leukemia, Type I *CBFβ*/*MYH11*, Inv(16)(p13.1q22), Case report

## Abstract

Inv(16)(p13.1q22) in acute myeloid leukemia (AML) is a common chromosomal abnormality. It leads to the core-binding factor ß-subunit (*CBFβ*)/smooth muscle myosin heavy chain 11 (*MYH11*) fusion gene. Different breakpoints were observed in the *CBFβ* gene at 16q22 and the *MYH11* gene at 16p13.1. For this reason, different *CBFβ*/*MYH11* fusion genes are generated, with more than 13 types having been reported to date. Type I *CBFβ*/*MYH11* fusion transcripts are very rare, with only 10 cases being reported to date. This case report describes a primary AML patient with inv(16)(p13.1q22) and a rare type I *CBFβ*/*MYH11* fusion gene. The morphological analysis did not conform to the typical M4eo. Abnormal eosinophils were less than 5%, and there was obvious dysgranulopoiesis. The patient was in hematological and genetic remission for 487 days after the initial chemotherapy cycles. However, the *CBFβ*/*MYH11* fusion had been constantly positive. Moreover, the presence of non-type A fusions may affect its biology and clinical prognosis. Therefore, further studies on understanding its biological and prognostic significance are essential.

## Introduction

Acute myeloid leukemia (AML) is a malignant clonal disease of hematopoietic stem/progenitor cells (HSPCs). The inv(16)(p13.1q22)/t(16;16)(p13.1;q22), *CBFβ*/*MYH11* AML is an independent subtype in the WHO classification of AML. The inv(16)/t(16;16) produces *CBFβ*/*MYH11* fusion gene, formed by the juxtaposition of a 5' sequence from *CBFβ* at 16q22 and the 3' sequence from *MYH11* at 16p13.1. The fusion sites between the *CBFβ* and *MYH11* genes usually differ, which results in the generation of different *CBFβ*/*MYH11* transcripts. To date, at least thirteen different fusion transcripts have been reported (1-119). The three most reported types include type A (79-87%), type E (5-9%), and type D (3-10%) (3,4). The remaining types have only been reported in case studies. The type I *CBFβ*/*MYH11* fusion transcript has rarely been described, and to our knowledge, only 10 cases have been reported (1-7). This report describes a *de novo* AML patient with inv(16)(p13.1q22) showing a rare type I *CBFβ*/*MYH11* fusion transcript.

## Case Report

A 50-year-old male farmer complained of fatigue and chest tightness with no obvious cause since March 20, 2019. After resting, his condition improved slightly, but then gradually worsened. The patient visited a local hospital for diagnosis on April 2, 2019. Peripheral blood film (PBF) showed white blood cells (WBC) of 5.6×10^9^/L, red blood cells (RBC) of 1.43×10^12^/L, hemoglobin (Hb) of 53.2 g/L, and platelets (PLT) of 8×10^9^/L. The attending physician recommended a transfer to a tertiary hospital for diagnosis and treatment. The patient was transferred to our hospital on April 4, 2019. Physical examinations showed enlarged bilateral submandibular lymph nodes without hepatosplenomegaly. PBF indicated WBC of 4.20×10^9^/L with 43% neutrophils, 26% lymphocytes, 26% monocytes, and 5% blasts. PBF also revealed RBC of 1.12×10^12^/L with anisocytosis, Hb of 41 g/L, and PLT of 6×10^9^/L. Bone marrow (BM) aspirates revealed a significant increase in bone marrow cellularity; 18% were myeloblasts with regular or irregular nuclei, fine chromatin, visible nucleoli, and reduced blue plasma. Nuclear-cytoplasmic dyssynchrony, nuclear malformations, binuclear, and Pelger malformed granulocytes accounted for 13% of the granulocytes, and 3% abnormal eosinophils were observed. Nucleated red blood cells were significantly reduced and accounted for 1.5%. Petal-nucleated red blood cells and anisocytosis were also observed; 18% of the aspirates were monocytes. Two granular megakaryocytes were observed on the whole slide, and the number of platelets was significantly reduced. Results from the bone marrow biopsy showed hypercellularity of the bone marrow tissue, with an increase in immature cells. Immunophenotyping of bone marrow cells showed 5.65% blasts, expressing CD34, CD117, CD13, HLA-DR, lack of CD7, CD10, CD38, CD33, CD11C, CD11b, CD64, CD15, CD19, CD123, CD56, CD36, CDCR4, CD14, CD300e, CD4, and CD2 (Supplementary Figure S1).

This study was approved by the Ethics Committee of the Traditional Chinese Medical Hospital of Langfang City, China.

### Conventional cytogenetics and fluorescent *in situ* hybridization

**Figure 1:**
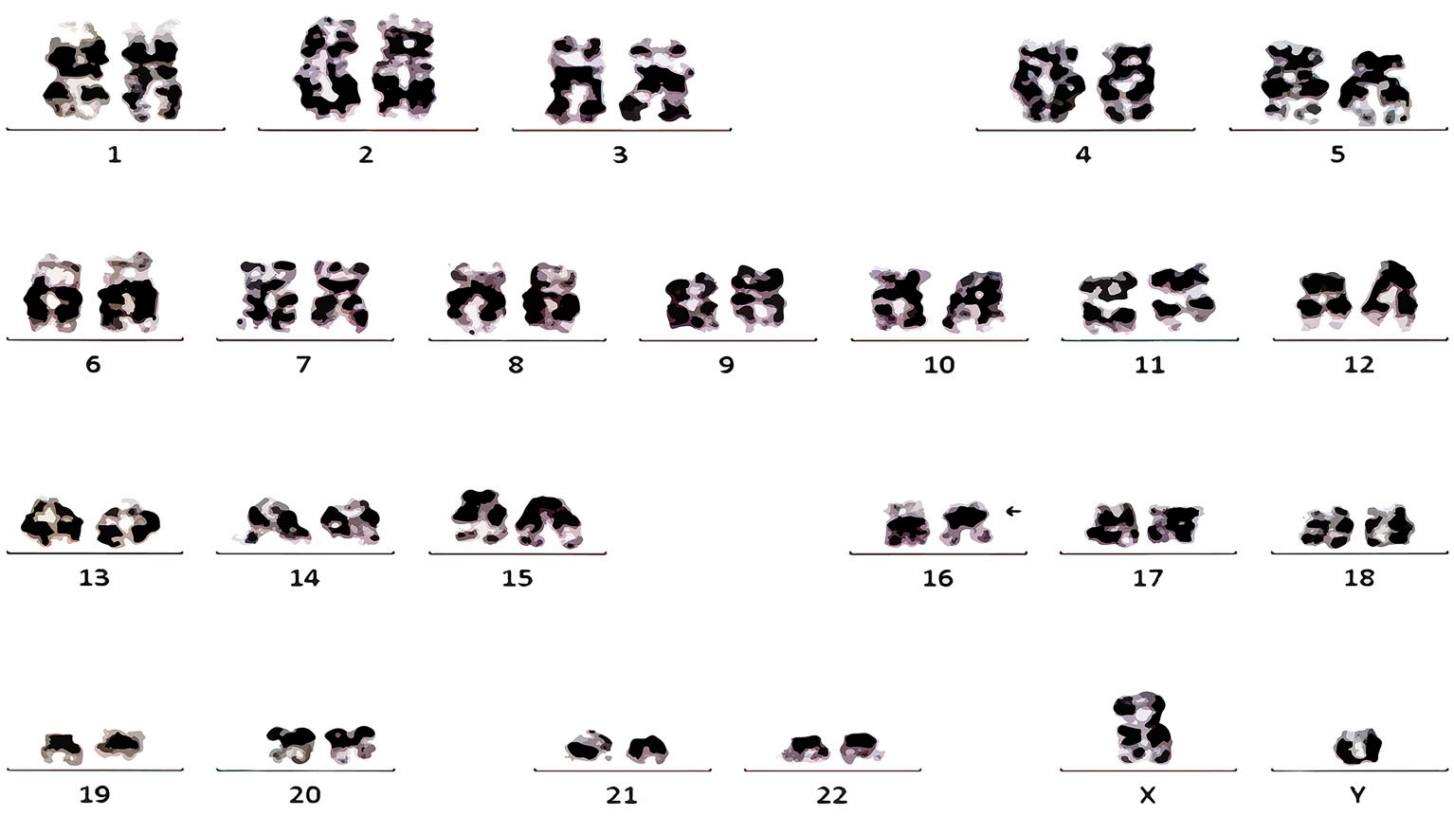
Representative karyotype showing 46,XY,inv(16)(p13.1q22)[10].

**Figure 2:**
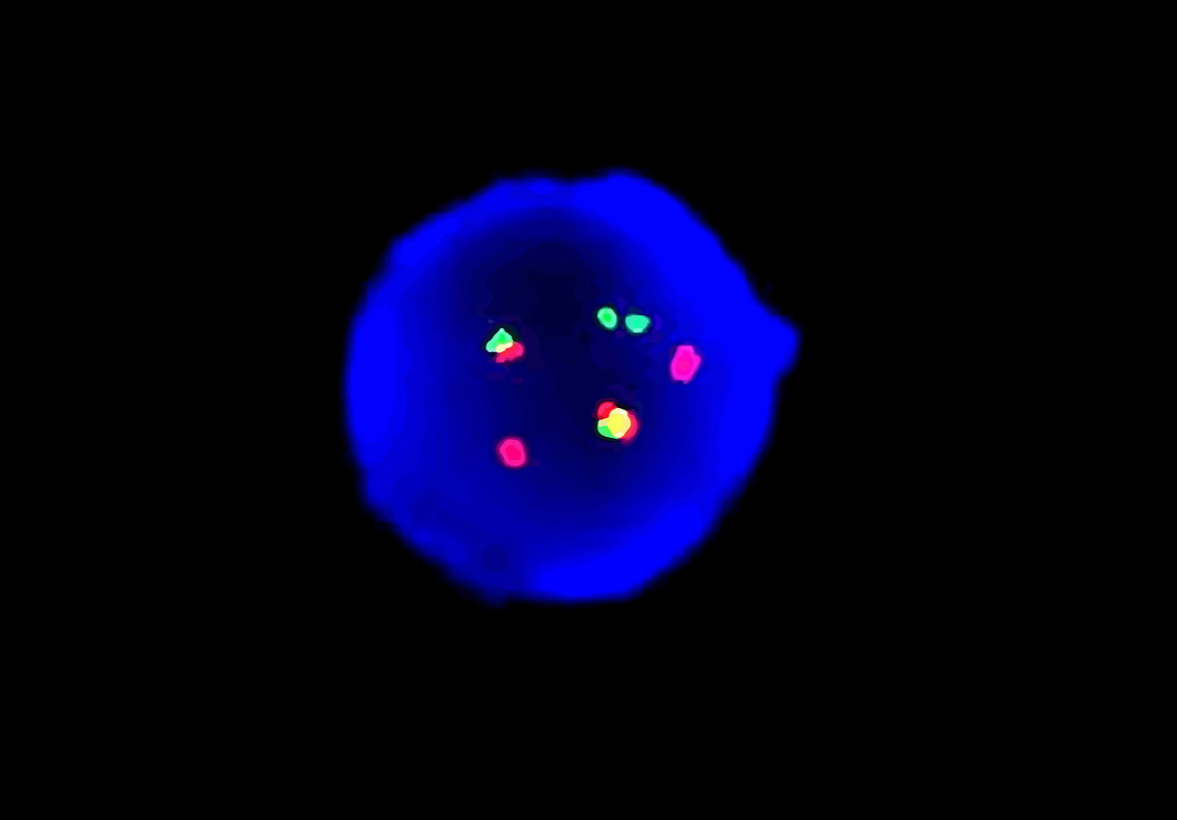
FISH result using the double color separation *CBFβ* probe (VYSIS), green fluorescent-labeled 3'*CBFβ* (16q22) probe, and red fluorescent-labeled 5'*CBFβ* probe. Split *CBFβ* abnormal signals on chromosome 16 (2R and 2G) are observed in interphase tetraploid cell nuclei.

Bone marrow nucleated cells (1-3×10^6^/mL) were cultured in Gibco bone marrow cell culture media (USA) for 48 h and then analyzed by G-banding based on an international system for Human Cytogenomic Nomenclature (ISCN 2016). Karyotype analysis demonstrated 46,XY,inv(16)(p13.1q22)[10] (Figure 1). Fluorescence *in situ* hybridization (FISH) was performed using the Vysis dual-color separation *CBFβ* probe (Abbott, USA), green fluorescent labeled 3'*CBFβ* (16q22) probe, and the red fluorescent-labeled 5'*CBFβ* probe. The rearrangement of the *CBFβ* gene fusion was detected on chr16 (Figure 2).

### Molecular analysis

**Figure 3:**
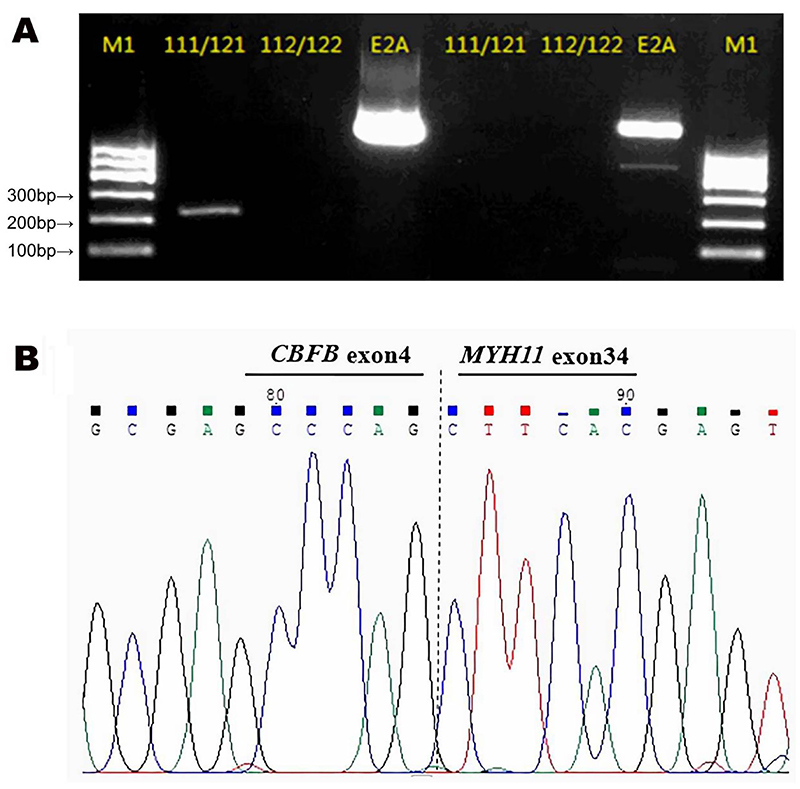
Gel electrophoresis and sequence analysis of the *CBFβ*/*MYH11* transcript. **A**, Gel electrophoresis 111/121 and 112/122 primer pairs used for amplification of the *CBFβ*/*MYH11* transcript. E2A is the internal reference primer. M1 is the DNA molecular weight marker 1 (100-600 bp, Tiangen, China). 111/121 amplified product showed a specific band between 200-300bp. **B**, Sequence analysis of the *CBFβ*/*MYH11* transcript showed a breakpoint between exon 4 of the *CBFβ* gene and exon 34 of the *MYH11* gene.

RT-PCR was used to detect the following gene fusions based on a previous study(20). All of these genes were negative: *BCR-ABL*, *SIL-TAL1*, *E2A-HLF*, *TEL-AML1*, *MLL-AF4*, *E2A-PBX1*, *AML1-ETO*, *MLL-AF9*, *PML-RARα*, *PLZF-RARα*, *STAT5b-RARα*, *MLL-AF6*, *MLL-AF10*, *MLL-ELL*, *MLL-ENL*, *NPM-MLF1*, *TEL-PDGFRB*, *FIP1L1-PDGFRA*, *AML1-MDS1*/*EVI1*, *DEK-CAN*, *TEL-ABL*, *ETV6-PDGFRA*, *NUP98-HoxA13*, *NUP98-HoxC11*, *NUP98-HoxD13*, *NUP98-HoxA9*, *NUP98- HoxA11*, *NUP98- PMX1*, *TEL-JAK2*, *MLL-AF17*, *MLL- AF1q*, *MLL- AF1p*, *MLL-AFX*, *MLL-SEPT6*, (*NPM, FIP1L1, PRKAR1A, NUMA1*)*-RARα*, *NPM-ALK*, *SET-CAN, TLS-ERG*, and *AML1-MTG16*. *WT1* was 11.7%. A rare fusion transcript of *CBFβ*/*MYH11* was detected, and electrophoresis of the PCR amplified product showed a positive band between 200-300bp (Figure 3A). Additional analysis of the PCR amplified product by capillary sequencing was performed. The sequence was as follows: TTTCAGAATTTTGAAGGCTCCCATGATTCTGAATGGAGTCTGTGTTATCTGGAAAGGCTGGATTGATCTCCAAAGACTGGATGGTATGGGCTGTCTGGAGTTTGATGAGGAGCGAGCCCAGCTTCACGAGTATGAGACGGAACTGGAAGACGAGCGAAAGCAACGTGCCCTGGCAGCTGCAGCAAAGAAGAAGCTGGAAGGGGACCTCTTCTAAA.

G denoted the end of the *CBFβ* exon4 and C denoted the beginning of the *MYH11* exon34 according to NM_002474.3 and CCDS_10565.1. This confirmed that *CBFβ* exon4 and *MYH11* exon34 were fused to form the fusion transcript. Comparing the CCDS sequence of the NCBI database, the *CBFβ*/*MYH11* fusion transcript belonged to a rare type I fusion transcript (Figure 3B) (6,10,12). The patient was then diagnosed with inv(16)(p13.1q22); *CBFβ*/*MYH11* (type I) AML. Next-generation sequencing (NGS) analysis was used to detect additional prognostic mutational genes, including *FLT3*, *NPM1*, *KIT*, *CEBPA*, *DNMT3A*, *IDH1*, *IDH2*, *TET2*, *EZH2*, *AML1*, *ASXL1*, *PHF6*, *TP53*, *SF3B1*, *SRSF2*, *U2AF1*, *ZRSR2*, *NRAS*, *CBL*, *SETBP1*, *ETV6*, and *JAK2*. No mutations in these genes were observed.

### Treatment and response

The patient underwent chemotherapy with decitabine (DAC), daunorubicin (DNR), and cytarabine (Ara-C) (DAC: 50 mg qod×3; DNR: 40 mg d1-2, 60 mg d3; Ara-C: 200 mg d1-4, 150 mg d5, 200 mg d6-7). In addition, supportive treatment including heart, liver, and stomach protection, antiemetics, RBC, and platelet transfusions, was administered. On the 24th day after chemotherapy, BM cellularity was reduced, hence, supportive treatment was continued. On the 41st day, the cellular composition of the BM aspirates consisted of 10% myeloblasts, 11% monoblasts and pre-monocytes, and 2% abnormal eosinophils. The patient was administered a second cycle of chemotherapy with DAC, homoharringtonine (HHT), Ara-C, and etoposide (VP-16) (DAC: 50 mg qod×3; HHT: 2mg d1-5, 3mg d6-7; Ara-C: 150mg d1-7; VP16 0.1g, d1-7). In addition, supportive treatment was also administered. Seventy-five days after the initial chemotherapy, BM morphology returned to normal, and chromosomes were 46,XY(20). FISH showed *CBFβ* gene separation and rearrangement. Chemotherapy was continued, and 272 days after the initial chemotherapy, *CBFβ* gene separation and rearrangement was negative as determined by FISH, and the *CBFβ*/*MYH11* fusion transcript showed positive. After 487 days of the initial chemotherapy, bone marrow morphology showed complete response (CR), with 46,XY(20) chromosomes. FISH was negative for *CBFβ* gene separation and rearrangement. However, the *CBFβ*/*MYH11* fusion transcript was still observed. At present, the patient is still undergoing regular chemotherapy and follow-up.

## Discussion

*CBFβ*/*MYH11* fusions mainly manifest from inv(16)(p13.1q22), and at much lower levels with t(16;16)(p13.1;q22)(5,8). *CBFβ*/*MYH11* fusions account for 5.04% of primary AML. The incidence of *CBFβ*/*MYH11* fusions in infants and children decreases with age, while the fusions increase steadily with age in adults. Fusions are rare in patients 50 years of age and over (20).

*CBFβ*/*MYH11* fusion transcripts are heterogeneous and depend on the different intron breakpoints between the exons of *CBFβ* and *MYH11* genes. To date, over 13 types of fusions have been reported, the majority of which being type A transcripts, with fewer D and E types, while the other types are rare. Type I fusion transcripts are very rare, with only 10 cases reported in the literature to date. Of the 10 cases, only 6 cases have been reported in detail (1,2,5-7).

We treated a primary AML patient with a rare type I fusion transcript. Morphological analysis did not conform to the typical M4eo. Abnormal eosinophils were less than 5%, and there was obvious dysgranulopoiesis. Chromosome karyotype analysis and FISH assays confirmed the presence of inv(16)(p13.1q22). Gene sequencing showed a type I *CBFβ*/*MYH11* fusion transcript. Type I, also known as type S/I, was first reported by Dissing et al. (1), followed by other cases (2-7). Inv(16)(p13.1q22)/t(16;16)(p13.1;q22) AML with non-type A *CBFβ*/*MYH11* fusion transcripts are more common in t-AML patients. Its occurrence has been associated with exposure to topoisomerase II inhibitors/topoisomerase I inhibitors (1,3,6,8). The other fusions that are rarer show a more atypical cytomorphology, mostly with pathologic eosinophils <5% and lower WBC counts (3,4,9). Atypical changes in chromosome numbers have been reported in the literature with chromosomes 8, 21, and 22. Several reports have suggested that the numerical gains in chromosomes 8, 21, and 22 occur mostly in patients with type A rather than rare fusion types (3). However, it has been reported that non-type A patients frequently have extra +8 and +21 chromosomes, with none having extra +22 chromosomes (4). No additional cytogenetic abnormalities and leukopenia were detected at the initial diagnosis in our patient. However, there were obvious abnormalities in granulocyte morphology. Among the reports published in patients with type I, there were 3 cases with t-AML and 5 cases with *de novo* AML (1,2,4-7).

The patient in this study had *de novo* AML. This suggests that type I is more common in patients with *de novo* AML. The type of *CBFβ*/*MYH11* fusion transcript is not an independent prognostic factor. No significant differences in overall survival (OS) or event-free survival (EFS) were observed with the type of fusion (3). Previous studies observed no significant differences in CR rate, the cumulative incidence of relapse (CIR), and OS between non-type A patients and type A patients. However, non-type A patients had longer EFS compared to type A patients. This may be related to the presence of KIT mutations rather than the type of fusion transcript. KIT mutations were observed in 24% of type A patients and none in non-type A patients (4). In addition, previous case reports showed that non-type A patients had a high CR rate and a better prognosis (2,5,9,12,13,16). In our patient, next-generation sequencing showed no genetic mutations related to prognosis. After a limited number of chemotherapy cycles, the patient was in hematological and genetic remission 487 days after the initial chemotherapy. However, the *CBFβ*/*MYH11* fusion had been always positive. The patient is currently undergoing regular chemotherapy and follow-up.

The limitation of this study is that the interphase FISH specimens could not be saved due to a long experimental time, and metaphase FISH could not be performed.

It is currently believed that the type of fusion has no effect on the prognosis of patients with inv(16)(p13.1q22)/t(16;16)(p13.1;q22). However, the presence of non-type A fusions, related distinctive clinical and genetic characteristics, and unique gene expression profiles may affect its biology and clinical outcome. Due to the limited number of *CBFβ*/*MYH11* fusion types, understanding its biological and prognostic significance is challenging.

## Supplementary Material

Click here to view [pdf].
